# The resident pathobiont *Staphylococcus xylosus* in *Nfkbiz*-deficient skin accelerates spontaneous skin inflammation

**DOI:** 10.1038/s41598-017-05740-z

**Published:** 2017-07-24

**Authors:** Yeji Kim, Yong-Soo Lee, Jin-Young Yang, Su-Hyun Lee, Yun-Yong Park, Mi-Na Kweon

**Affiliations:** 1 Mucosal Immunology Laboratory, Department of Convergence Medicine, University of Ulsan College of Medicine/Asan Medical Center, Seoul, Korea; 2 ASAN Institute for Life Science, Asan Medical Center, Seoul, Korea

## Abstract

IκBζ, which is encoded by the *Nfkbiz* gene, is a member of the nuclear IκB family of proteins that act as transcriptional regulators via association with NF-κB. *Nfkbiz*-deficient (*Nfkbiz* ^−/−^) mice develop spontaneous dermatitis; however, the underlying mechanism has yet to be elucidated. In our study, we found higher skin pathology scores and more serum IgE antibodies and trans-epidermal water loss in *Nfkbiz* ^−/−^ than in *Nfkbiz*-sufficient (*Nfkbiz* ^+/−^) mice. There was also greater expansion of IFN-γ-, IL-17A-, and IL-22-secreting CD4^+^ T cells and of IL-17A-secreting γδ^+^ T cells in the skin of *Nfkbiz* ^−/−^ mice than in with *Nfkbiz* ^+/−^ mice. Pyrosequencing analysis showed decreased diversity of resident bacteria and markedly expanded *Staphylococcus (S.) xylosus* in the skin of *Nfkbiz* ^−/−^ mice. Oral administration of antibiotics including cephalexin and enrofloxacin ameliorated skin inflammation. Topical application of *S. xylosus* also resulted in the expansion of IL-17A-secreting CD4^+^ T cells along with high levels of pro-inflammatory cytokines and chemokines in the skin of *Nfkbiz* ^−/−^ mice. The expansion of commensal *S. xylosus* may be one cause of skin dysbiosis in *Nfkbiz* ^−/−^ mice and suggests that the *Nfkbiz* gene may play a regulatory role in the microbiota-skin immunity axis.

## Introduction

The skin acts as an important physical barrier that is directly exposed to numerous pathogens in the environment. It maintains a symbiotic relationship with various commensal microorganisms that inhabit it^1^. Changes in microbial composition have been found in many skin diseases including atopic dermatitis (AD) and psoriasis^2, 3^. For instance, microbiome analysis in patients with AD revealed that the proportion of *Staphylococcus (S.) aureus* that increased during disease flares correlated with disease severity^2^. *S. aureus* was also isolated from skin lesions of STAT3-deficient mice and of ADAM17-deficient mice with spontaneous dermatitis, and antibiotic treatments were essential to control the disease progression^4, 5^. *S. xylosus* is a coagulase-negative gram-positive *staphylococcal* species and a common skin commensal bacterium of humans and other mammals^6^. Some cases of *S. xylosus* infection, including erythema nodosum^7^, pyelonephritis^8^, and corneal infections^9^ occur in humans. In laboratory mice, *S. xylosus* has been associated with spontaneous dermatitis in both NOS2^−/−^ mice^10^ and athymic nude mice^11^ and with conjunctivitis in NADPH oxidase^−/−^ mice^12^, implying that *S. xylosus* is an opportunistic pathogen. Although microorganisms are not the only inducers of skin disease, it seems likely that microbial alteration and the predominance of specific resident bacteria directly affect dermatitis severity.

As gut microbes are associated with gut immunity, commensal microbiota in the skin also play a pivotal role in local immunity to maintain homeostasis both in a steady state and during infection^13^. When the skin of germ-free mice was colonized with *S. epidermidis* and compared with that of conventional mice, the germ-free mice had fewer resident-effector T cells than the conventional mice and developed protective T-cell responses against the *Leishmania major* parasite^14^. In addition, *S. epidermidis*-induced IL-17A^+^CD8^+^ T cells enhanced innate barrier immunity and ameliorated pathogen infection^15^. However, when the expansion and aberration of commensal microbiota with pathogenic potential occurred under specific circumstances, such as infection and injury, it could disturb immune system homeostasis and worsen inflammation. Previous studies revealed that staphylococcal enterotoxin B or alpha-toxin derived by *S. aureus* induced IL-17A secretion, leading to the abnormal proliferation of keratinocytes and recruitment of inflammatory cells^16, 17^. Notably, patients with severe skin signs showed more *S. aureus* specific-IgE antibodies (Abs) than those with mild symptoms^18^. Because of the complex interplay among commensal microbes, immunity, and environmental factors in the skin mucosa, the underlying mechanisms have yet to be elucidated.

IκBζ, encoded by the *Nfkbiz* gene, is a member of the nuclear IκB family of proteins that act as transcriptional regulators via association with NF-κB. Several previous studies revealed a critical role of IκBζ signaling in the regulation of immune responses^19–22^. IκBζ^−/−^ mice showed severe skin irritation in the face, neck, and periocular regions and several signs of Sjögren’s syndrome^23, 24^. IκBζ interacts with NF-κB and is associated with both positive and negative regulation of NF-kB transcriptional activity. Like other IκB proteins, it has inhibitory effects on the transcription of inflammatory genes regulated by NF-kB, such as TNF-α, but it can also induce pro-inflammatory cytokines, such as IL-6 and IL-12p40^25^. IκBζ also plays an important role in adaptive immunity by regulating Th17 development^19^. Although IL-6 and IL-17A production was significantly reduced in IκBζ-deficient cells *in vitro* ^19, 23^, highly elevated levels of these cytokines were detected *in vivo* ^26^. It is known that IκBζ deficiency in hematopoietic cells is not the major cause of spontaneous inflammation^24^. The specific depletion of IκBζ in lymphocytes or myeloid cells does not induce inflammation in the conjunctiva and ocular skin, although enhanced apoptosis in IκBζ-deficient epithelial cells is involved in the onset of Sjögren’s syndrome-like autoimmune disease in IκBζ^−/−^ mice^24^. It is not clear how the immune system is overactivated by depletion of IκBζ.

In this study, we demonstrated that *Nfkbiz*-deficient (*Nfkbiz* ^−/−^) mice with atopic-like dermatitis have elevated serum IgE Abs and drastically increased numbers of IL-17A-secreting CD4^+^ T cells in skin. Microbiome analysis showed the predominant expansion of *Staphylococcus* sequences, particularly *S*. *xylosus*, and a significant decrease in the diversity of commensal bacteria. Antibiotics completely eliminated dermatitis symptoms, implying specific taxonomic alterations associated with the induction of hyper-immune responses in the skin. Of note, the inoculation of *S*. *xylosus* in the skin of *Nfkbiz* ^−/−^ and their heterozygous littermates (*Nfkbiz* ^+/−^) resulted in the expansion of IL-17A production from CD4^+^ T cells.

## Results

### *Nfkbiz*^−/−^ mice developed spontaneous skin inflammation

**Figure 1:**
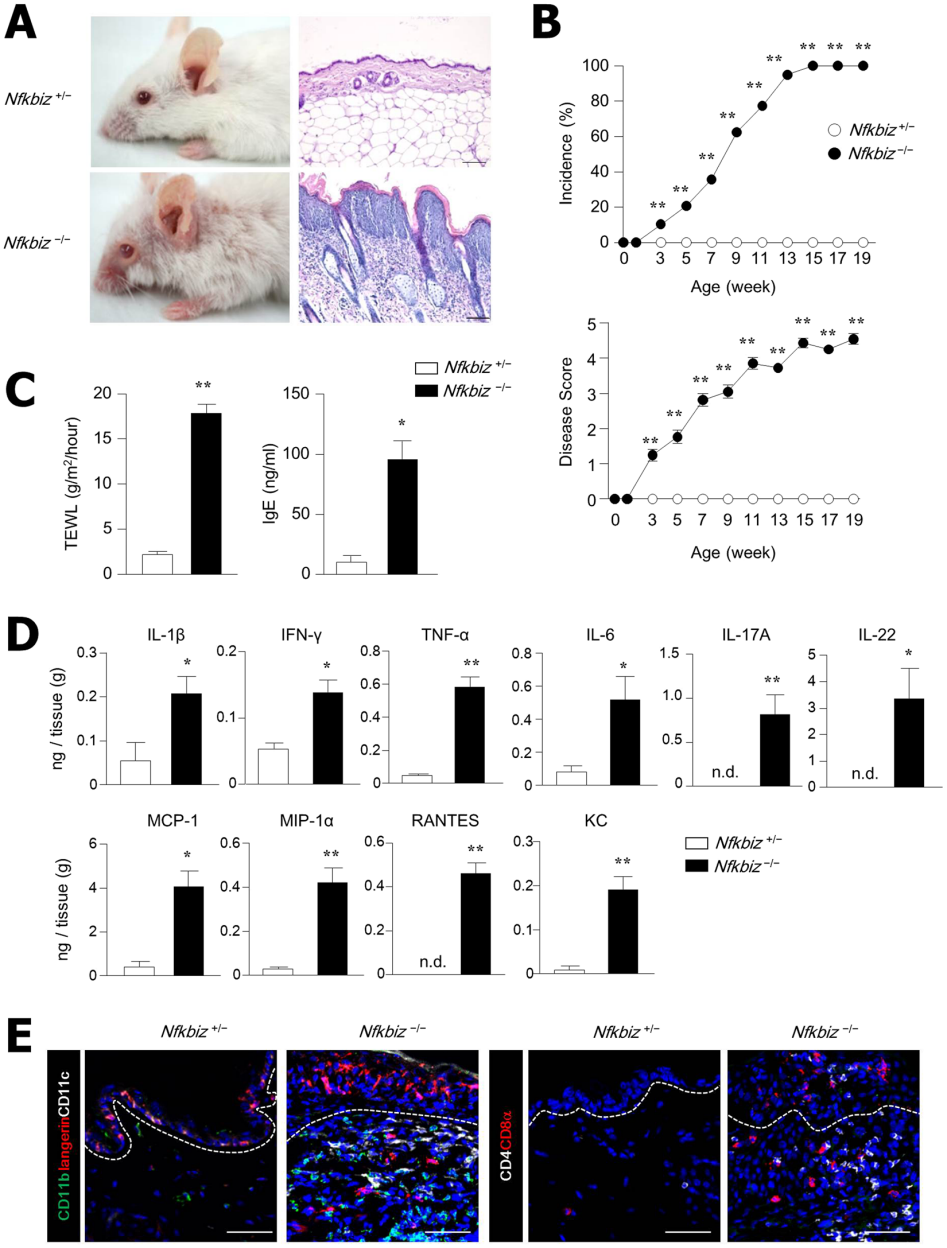
Spontaneous skin inflammation develops in *Nfkbiz* ^−/−^ mice. (**A**) Representative skin phenotype and histology of skin biopsy from *Nfkbz* ^+/−^ and *Nfkbiz* ^−/−^ mice at age 10 weeks. Scale bar = 50 µm. (**B**) Disease incidence and progression of dermatitis in *Nfkbiz* ^+/−^ and *Nfkbiz* ^−/−^ mice (n = 20). Dermatitis incidence (score ≥ 3) is shown as a percentage. (**C**) Trans-epidermal water loss (TEWL) and serum IgE antibody levels in *Nfkbiz* ^+/−^ and *Nfkbiz* ^−/−^ mice. (**D**) Cytokines and chemokine levels in a skin homogenate. (**E**) Confocal microscopy of skin biopsies from *Nfkbiz* ^+/−^ and *Nfkbiz* ^−/−^ mice. Scale bar = 50 μm. Data are representative of three independent experiments. All data are mean ± s.e.m. *p < 0.05, **p < 0.01.

To clarify the mechanism underlying the development of dermatitis in *Nfkbiz* ^−/−^ mice, we first confirmed the disease progression in our facility. As reported by others^23^, we established that erosions and hair loss that were exhibited throughout the ocular region beginning about age 4 weeks in *Nfkbiz* ^−/−^ mice then extended to the whole body (Fig. 1A). In these mice, disease incidence and clinical score were also significantly increased from about age 4 weeks in *Nfkbiz* ^−/−^ mice (Fig. 1B). The levels of transepidermal water loss (TEWL), which show abnormal skin barrier function, and of serum IgE Ab, which has a positive correlation with the severity of atopic dermatitis and psoriasis^27, 28^, was higher in the *Nfkbiz* ^−/−^ mice than in *Nfkbiz*-sufficient (*Nfkbiz* ^+/−^) mice (Fig. 1C). In addition, the levels of various pro-inflammatory cytokines (i.e., IL-1β, IFN-γ, TNF-α, IL-6, IL-17A, and IL-22) and chemokines (i.e., MCP-1, MIP-1α, RANTES, and KC) were significantly increased in the serum of *Nfkbiz* ^−/−^ mice when compared with *Nfkbiz* ^+/−^ mice (Fig. 1D). There were marked increases in the numbers of CD11b^+^ or CD207^+^ or CD11c^+^ cells and CD4^+^ or CD8α^+^ cells infiltrating into the epidermis and dermis of *Nfkbiz* ^−/−^ mice as determined by confocal microscopy analysis (Fig. 1E). We conducted an intense analysis and found no significant phenotype differences in the innate lymphoid cell subsets isolated from *Nfkbiz* ^+/−^ and *Nfkbiz* ^−/−^ mice (data not shown). Once we confirmed the development of dermatitis in *Nfkbiz* ^−/−^ mice, we performed further experiments.

### IL-17A- and IL-22-secreting skin-resident T cells increased in *Nfkbiz*^−/−^ mice

**Figure 2:**
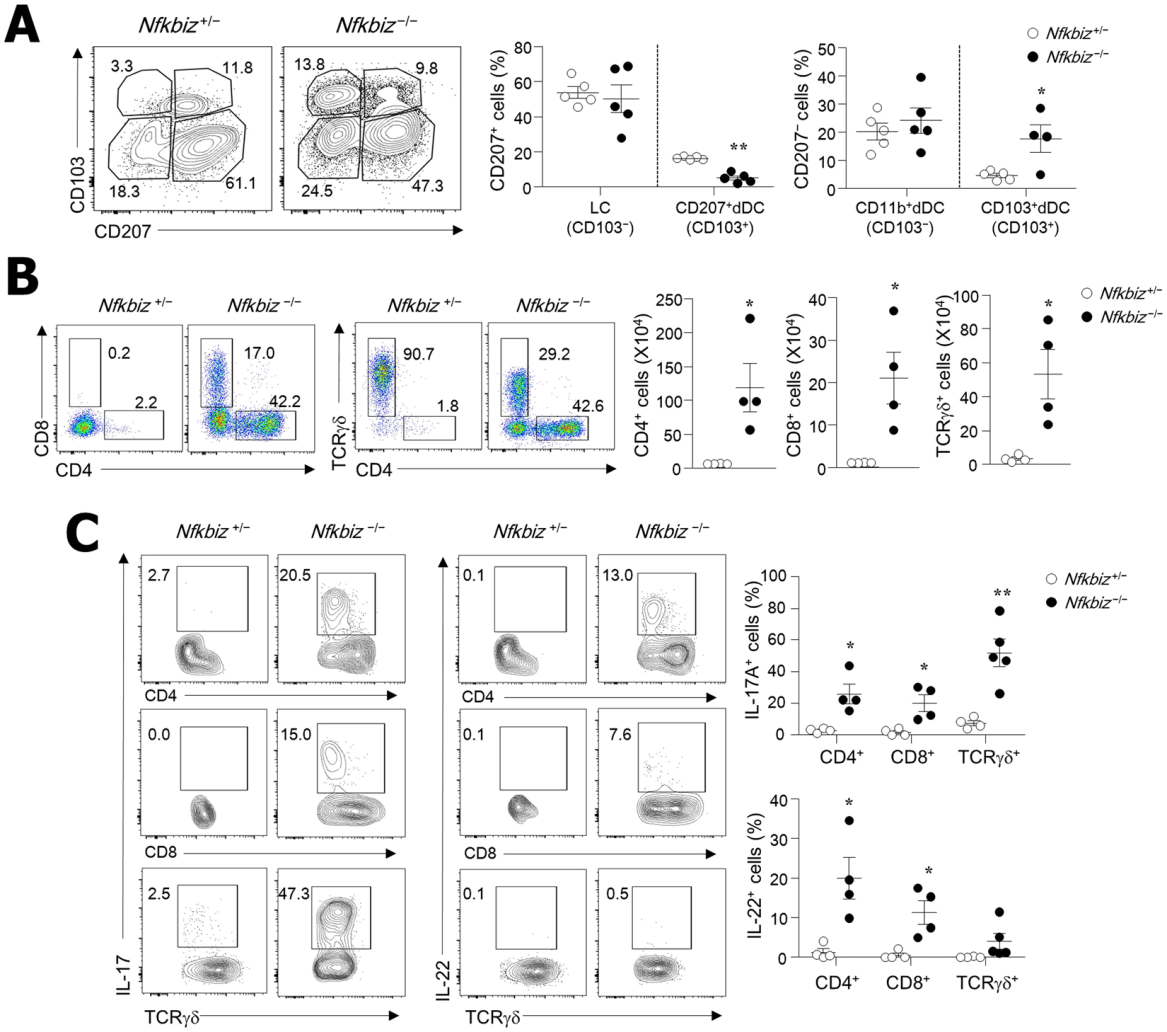
IL-17A- and IL-22-secreting T cells infiltrate into skin of *Nfkbiz* ^−/−^ mice. Flow cytometry analysis of (**A**) DC subsets in CD45^+^MHCII^+^CD11c^+^ cells and (**B**) T cells in skin of *Nfkbiz* ^+/−^ and *Nfkbiz* ^−/−^ mice. (**C**) IL-17A and IL-22 production from each cell subset after gating CD45-positive skin cells. Data are representative of three independent experiments. *p < 0.05, **p < 0.01.

To determine the cell types involved in dermatitis in *Nfkbiz* ^−/−^ mice, we analyzed immune cells in the skin via flow cytometry. The skin-resident dendritic cells (DCs) include Langerhans cells (LCs) in the epidermis and DCs in the dermis (dDCs), which can be divided into various subsets^29^. Absolute numbers of MHCII^+^CD11c^+^ cells were highly elevated in the epidermis and dermis of *Nfkbiz* ^−/−^ mice in comparison with those in *Nfkbiz* ^+/−^ mice (Fig. S1A). Among four DC subsets in skin, the relative percentages of CD103^+^ dDCs (CD11b^+/−^CD207^−^CD103^+^) were increased while CD207^+^ dDCs (CD11b^+^CD207^+^CD103^+^) were decreased in *Nfkbiz* ^−/−^ mice when compared with *Nfkbiz* ^+/−^ mice (Fig. 2A). No significant changes of relative percentages were found in LCs (CD11b^+^CD207^+^CD103^−^) and CD11b^+^ dDCs (CD11b^+^CD207^−^CD103^−^) between *Nfkbiz* ^−/−^ and *Nfkbiz* ^+/−^ mice (Fig. 2A). We next addressed skin-resident T-cell subsets and found that γδ^high^ T cells with few CD4 and CD8 T cells were present in the skin of *Nfkbiz* ^+/−^ mice, but *Nfkbiz* ^−/−^ mice with AD had massive infiltration of CD4, CD8, and γδ^mid^ T cells (Fig. 2B). Interestingly, accumulated CD4, CD8, and γδ^mid^ skin-resident T cells obtained from *Nfkbiz* ^−/−^ mice predominantly produced IL-17A and IL-22 (Fig. 2C). When these findings are considered together, CD103^+^ dDCs and IL-17A- and IL-22-secreting skin-resident T cells may be associated with skin inflammation in *Nfkbiz* ^−/−^ mice.

### *Nfkbiz*-deficient skin exhibited decreased diversity of the bacterial community and an increase in *S. xylosus*

**Figure 3:**
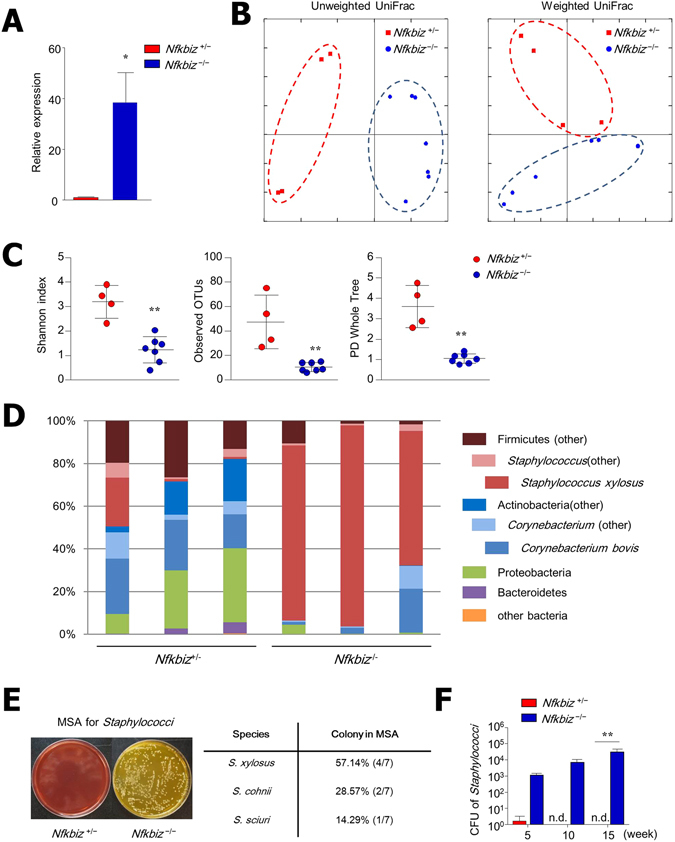
Increased bacterial burden and decreased bacterial diversity in skin of *Nfkbiz* ^−/−^ mice. (**A**) Relative expression of 16 s rDNA universal bacteria in skin of *Nfkbiz* ^+/−^ and *Nfkbiz* ^−/−^ mice. (**B**) Principal coordinate analysis of unweighted and weighted UniFrac distances between samples from skin of *Nfkbiz* ^+/−^ and *Nfkbiz* ^−/−^ mice. (**C**) Shannon index, observed operational taxonomic units (OTUs), and phylogenetic diversity (PD) whole tree of *Nfkbiz* ^+/−^ and *Nfkbiz* ^−/−^ mice. (**D**) Composition of skin bacterial communities in *Nfkbiz* ^+/−^ and *Nfkbiz* ^−/−^ mice at phylum level (>1% of total 16S rRNA sequences) with additional speciation of *Corynebacterium* and *Staphylococcus* genera. Each bar represents relative abundance in one mouse. Results are representative of two experiments (n = 4–6 per group). (**E** and **F**) Quantification of *Staphylococcus* species from skin swabs of *Nfkbiz* ^+/−^ and *Nfkbiz* ^−/−^ mice performed using mannitol salt agar (MSA). Data are mean ± s.e.m. (**A** and **F**). *p < 0.05, **p < 0.01. n.d., not detected.

Numerous studies have shown that dysbiosis of the skin microbiome is associated with development of AD and psoriasis in human patients and in animal models^2, 3, 5^. Hence, we next addressed the skin microbiota to determine whether the resident microbiota is also related to the development of dermatitis in *Nfkbiz* ^−/−^ mice. When quantity of bacterial 16S rRNA was assessed by PCR, there was a significantly higher bacterial burden in the skin homogenates of *Nfkbiz* ^−/−^ mice than from *Nfkbiz* ^+/−^ mice (Fig. 3A). Further pyrosequencing analysis using skin tissue homogenates revealed that *Nfkbiz* ^+/−^ and *Nfkbiz* ^−/−^ mice have segregated microbiota composition (Fig. 3B) and that *Nfkbiz* ^−/−^ mice have less diversity and species richness in than *Nfkbiz* ^+/−^ mice (Fig. 3C). The *Nfkbiz* ^−/−^ mice had decreased levels of *Pseudomonas*, *Acinetobacter*, *Gemella*, *Ochrobactrum*, and *Rhodococcus* genera (data not shown) but high abundance of *S. xylosus* (Fig. 3D). To further confirm the expansion of *S. xylosus* under disease conditions, we next cultured skin swabs from *Nfkbiz* ^+/−^ and *Nfkbiz* ^−/−^ mice on mannitol salt agar (MSA), a medium selective for *Staphylococcus* ^30^. Consistent with the pyrosequencing data, numerous yellow colonies on MSA, mostly identified as *S. xylosus* by 16S rRNA sequencing, were obtained from skin swab culture of *Nfkbiz* ^−/−^ mice (Fig. 3E). In addition, elevated numbers of *Staphylococcus* colonies were detected with increasing age (Fig. 3F), which correlated with the clinical score of *Nfkbiz* ^−/−^ mice (Fig. 1B). Overall, decreased bacterial diversity with the striking expansion of *S. xylosus* occurred in the skin of *Nfkbiz* ^−/−^ mice with dermatitis.

### Antibiotic treatment ameliorated dermatitis in *Nfkbiz*^−/−^ mice

**Figure 4:**
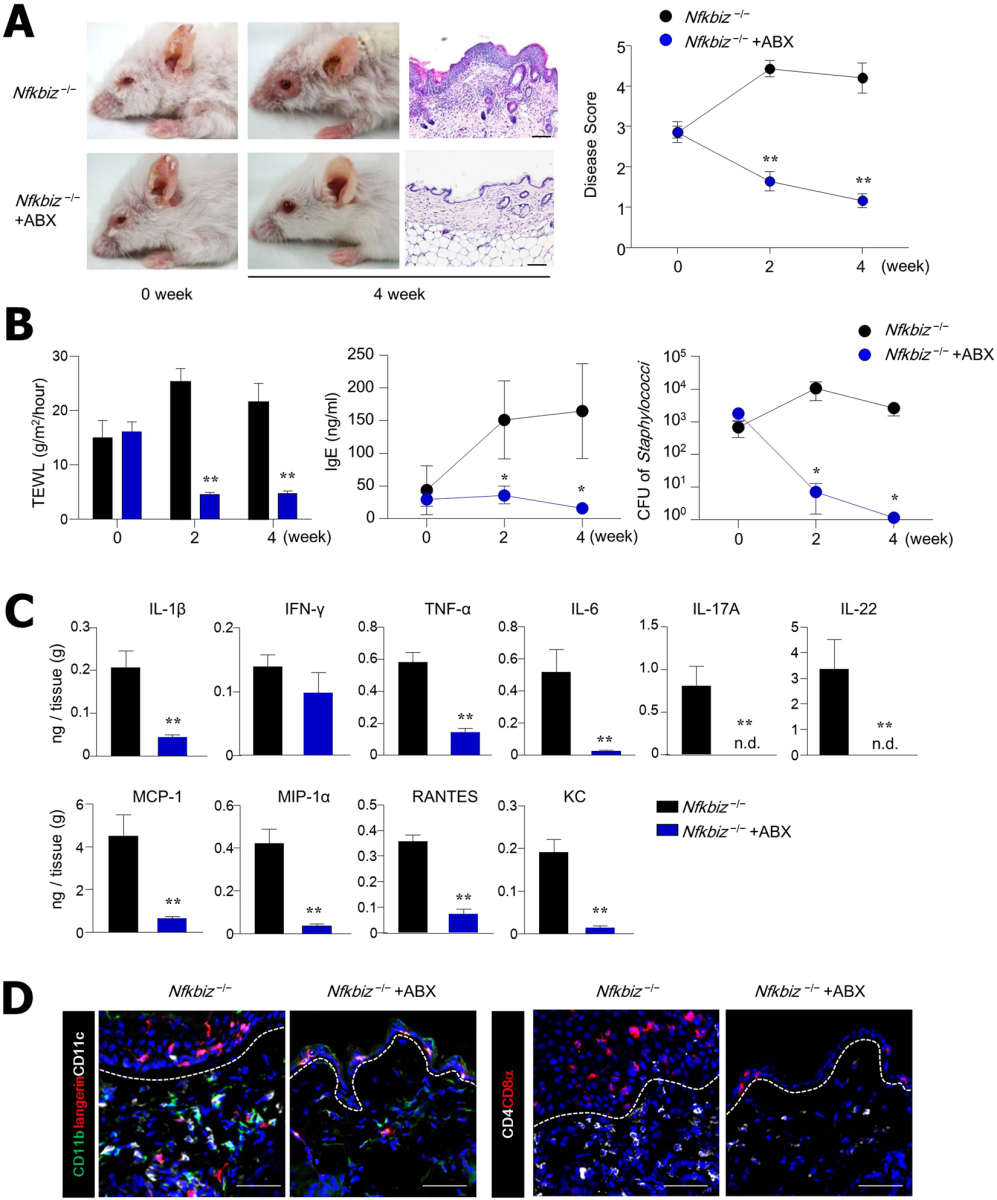
Antibiotic (ABX) treatment eliminates skin inflammation. (**A**) Representative skin phenotype and histology of skin biopsy from untreated (water alone) and ABX-treated *Nfkbiz* ^−/−^ mice at age 6 weeks. Scale bar = 50 μm. (**B**) TEWL, serum IgE levels, and colony-forming units (CFU) of *Staphylococcus* species. (**C**) Cytokine and chemokine levels in skin homogenate. (**D**) Confocal microscopy of skin biopsies. Scale bar = 50 μm. Data are representative of three independent experiments. All data are mean ± s.e.m. *p < 0.05, **p < 0.01.

To clarify the direct role of resident microbiota in controlling symbiosis in the skin, we next applied antibiotics to *Nfkbiz* ^−/−^ mice. Oral administration of antibiotics including cephalexin and enrofloxacin for 4 weeks significantly ameliorated the skin inflammation in *Nfkbiz* ^−/−^ mice, during which time the disease score of untreated mice worsened (Fig. 4A). To support this amelioration of skin inflammation, we found significantly lower levels of TEWL and IgE Abs in the antibiotic-treated *Nfkbiz* ^−/−^ mice than in the untreated *Nfkbiz* ^−/−^ mice (Fig. 4B). We also observed a decline in *Staphylococcus* species in the skin, indicating that the antibiotics effectively suppressed *S. xylosus* (Fig. 4B). The oral administration of antibiotics suppressed the levels of pro-inflammatory cytokines (i.e., IL-1β, TNF-α, IL-6, IL-17A, and IL-22) and chemokines (i.e., MCP-1, MIP-1α, RNATES, and KC) in the skin (Fig. 4C). In this regard, the numbers of infiltrated DCs and T cells were significantly diminished in the skin tissue of antibiotic-treated *Nfkbiz* ^−/−^ mice (Figs 4D and S1B).

**Figure 5:**
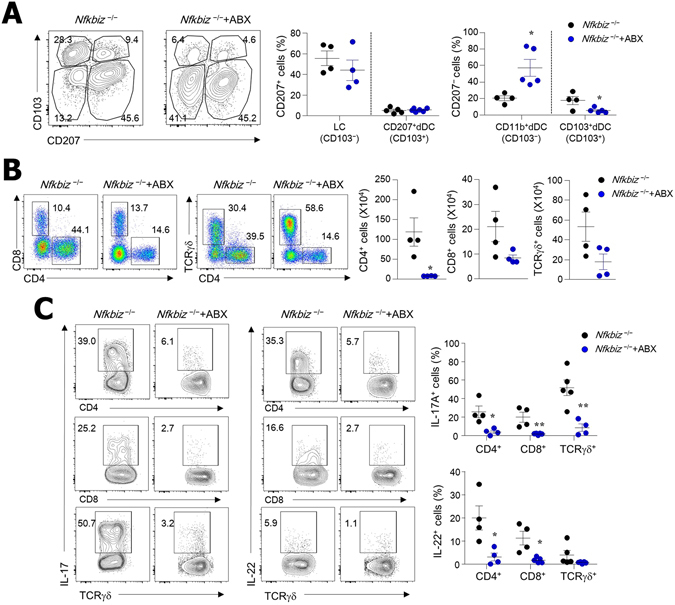
Infiltrated IL-17A- and IL-22-secreting T cells are reduced in skin of *Nfkbiz* ^−/−^ mice after ABX treatment. Flow cytometry analysis of (**A**) DCs in CD45^+^MHCII^+^CD11c^+^ cells and (**B**) T cells in CD45^+^ cells isolated from skin of water- and ABX-treated *Nfkbiz* ^−/−^ mice at age 6 weeks. (**C**) IL-17A and IL-22 production by CD45^+^ cells in skin. Data are representative of three independent experiments. *p < 0.05, **p < 0.01.

We further explored DC and T-cell subsets associated with skin inflammation induced by resident bacteria. Among the DC subsets, only CD103^+^ dDC subsets were significantly reduced by antibiotic treatments (Fig. 5A). The oral administration of antibiotics diminished total recovered numbers of CD4, CD8, and γδT cells and the percentages of those cells (Fig. 5B). Interestingly, IL-17A-secreting CD4, CD8, and γδT cells were significantly reduced by antibiotic treatments (Fig. 5C). Consistent with the ELISA data shown in Fig. 4C, antibiotic treatments were not associated with IFN-γ secretion by CD4^+^ and CD8^+^ T cells (Fig. S2). Collectively, these data suggest that the dominant expansion of *S. xylosus*, which inhabits the skin of mammals as a commensal bacterium, is one factor that contributes to the onset of skin inflammation in the absence of the *Nfkbiz* gene. Moreover, IL-17A and IL-22 are strongly related to the expansion of *S. xylosus*.

### Antibiotic treatment reduced gene expression related to skin inflammation of *Nfkbiz*^−/−^ mice

**Figure 6:**
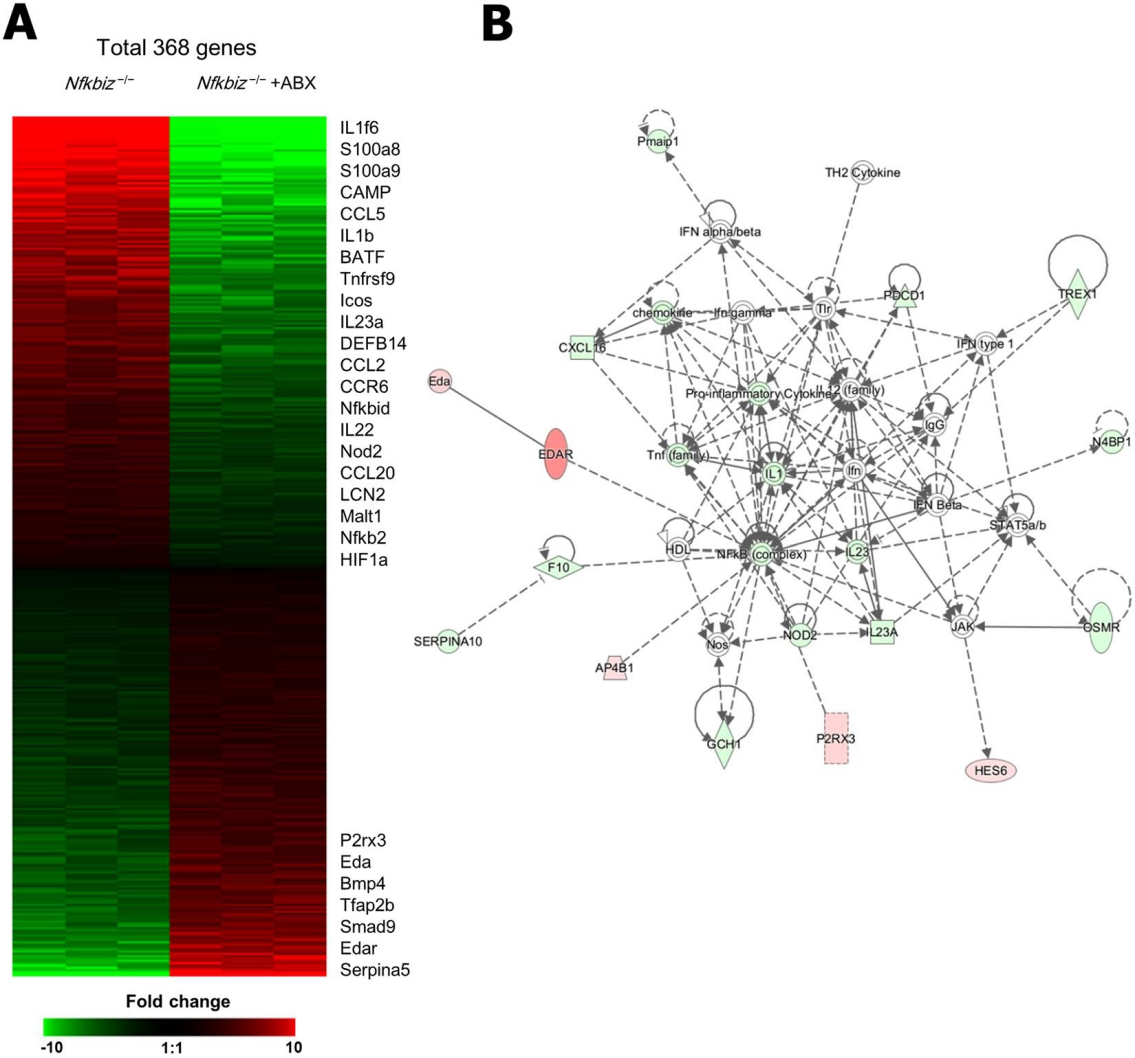
ABX treatment reduces gene expression related to inflammation in skin of *Nfkbiz* ^−/−^ mice. (**A**) Microarray analysis of skin from water- and ABX-treated *Nfkbiz* ^−/−^ mice (n = 3 per group). Genes with significantly changed expression (fold change > 1.0 and *p* < 0.05) after ABX treatment clustered in a heat map. (**B**) A gene interaction network, associated with the amelioration of spontaneous dermatitis in *Nfkbiz* ^−/−^ mice, identified by Ingenuity Pathway Analysis software. Solid lines indicate direct interaction; dashed lines indicate indirect interaction.

To investigate which genes are involved in the regulation of skin inflammation induced by resident bacteria in the absence of the *Nfkbiz* gene, we compared the gene expression profiles in the skin tissues of *Nfkbiz* ^+/−^ mice, *Nfkbiz* ^−/−^ mice, and antibiotic-treated *Nfkbiz* ^−/−^ mice. The expression levels of 694 and 368 genes were higher and lower (i.e., changed ≥ 2-fold) in *Nfkbiz* ^−/−^ mice than in *Nfkbiz* ^+/−^ mice (Fig. S3A) and in antibiotic-treated *Nfkbiz* ^−/−^ mice than in untreated *Nfkbiz* ^−/−^ mice (Fig. 6A), respectively. As expected, the gene expression levels of pro-inflammatory cytokines (i.e., IL-17A, IFN-γ, TNF, and IL-18) were higher in the skin of *Nfkbiz* ^−/−^ mice than in *Nfkbiz* ^+/−^ mice. The expression of the S100a9 gene, which is a marker of inflammation, was also significantly increased in the inflamed skin of *Nfkbiz* ^−/−^ mice (Fig. S3A). In contrast, the gene expression level of ectodysplasin A (Eda), which contributes to human and murine skin repair^31^, was lower in the skin of *Nfkbiz* ^−/−^ mice than in *Nfkbiz* ^+/−^ mice (Fig. S3A). Furthermore, IL-17A target genes (i.e., S100a9, S100a8, CAMP, and LCN2) were remarkably upregulated in the skin of *Nfkbiz* ^−/−^ mice with dermatitis (Fig. S3A). We next created and analyzed a hit gene network predicted by Ingenuity Pathway Analysis (IPA) software. Activation of the NF-kB signal pathway in the skin of *Nfkbiz* ^−/−^ mice was as predicted by the IPA software (Fig. S3B). Notably, the depletion of resident microbiota by antibiotic treatment resulted in the reduction of gene expression related to inflammation (i.e., S100a8, S100a9 and Tnfrsf9), but the activation of gene expression was associated with skin repair (i.e., Eda and Eda receptor) (Fig. 6A). Th17 cell-associated genes, (i.e., S100a8, CAMP, BATF, IL-23A, LCN2, and HIF-1α) highly expressed in *Nfkbiz* ^−/−^ mice with dermatitis, were downregulated after antibiotic treatments (Fig. 6A). By analysis with the IPA software, downregulation of the NF-kB signal pathway was predicted in the skin of *Nfkbiz* ^−/−^ mice in the absence of resident microbiota after antibiotic treatment, in comparison with that in untreated *Nfkbiz* ^−/−^ mice (Fig. 6B). Overall, these results support our conclusion that unusual expansion of the resident microbiota may be a key factor in the development of skin inflammation.

### Differentiation of Th17 cells in the skin after topical application of *S. xylosus*

**Figure 7:**
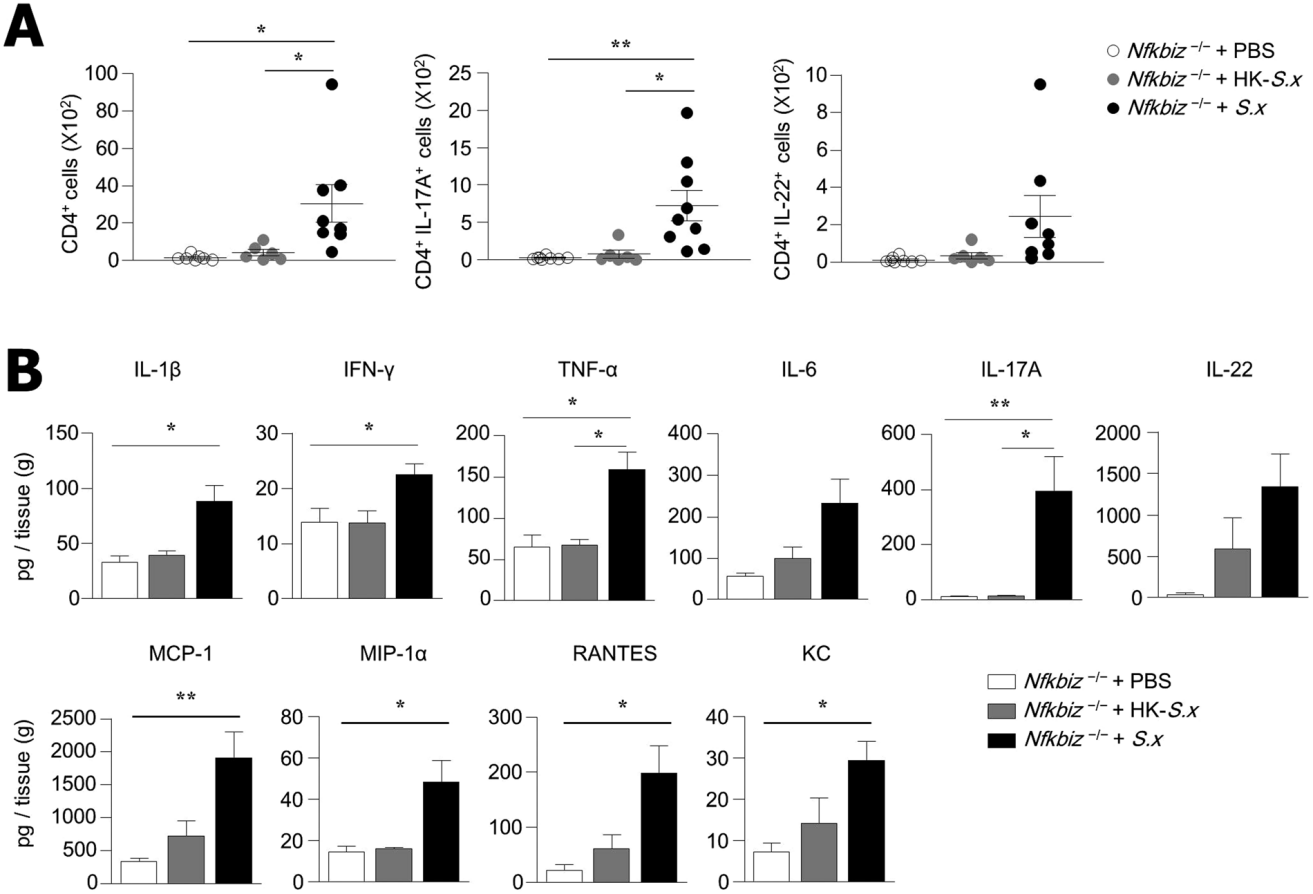
Topical application of *S. xylosus* leads to differentiation of Th17 cells in skin of *Nfkbiz* ^−/−^ mice. (**A**) Flow cytometry analysis of IL-17A and IL-22 production in CD4^+^ cells from skin of PBS, heat-killed *S. xylosus (HK-S.x)*, or *S. xylosus(S.x)*-inoculated *Nfkbiz* ^−/−^ mice. (**B**) Cytokine and chemokine levels in skin homogenate. Data are representative of three independent experiments. Data are mean ± s.e.m. *p < 0.05, **p < 0.01.

The amelioration of skin inflammation resulting from the administration of antibiotics prompted us to investigate whether the topical application of *S. xylosus* induces skin inflammation in *Nfkbiz* ^−/−^ mice. We pretreated *Nfkbiz* ^+/−^ and *Nfkbiz* ^−/−^ mice with antibiotics for 2 weeks to reduce resident commensals and topically applied *S. xylosus* to the back skin for 2 weeks. Topical inoculation resulted in the persistent colonization of *S. xylosus* on the skin of both *Nfkbiz* ^+/−^ and *Nfkbiz* ^−/−^ mice after antibiotic treatment (Fig. S4). The frequency of LCs and dDCs in the skin was not altered by *S. xylosus* inoculation (data not shown). Of interest, the topical application of *S. xylosus* induced the accumulation of CD4^+^ T cells in the skin and markedly enhanced the production of IL-17A in the skin of *Nfkbiz* ^+/−^ and *Nfkbiz* ^−/−^ mice (Figs 7A and S5A). There were many more IL-17A-secreting CD4^+^ T cells in the skin of *Nfkbiz* ^−/−^ mice than in *Nfkbiz* ^+/−^ mice. The numbers of IL-22-secreting CD4^+^ T cells were only slightly increased by *S. xylosus* application, which did not reach statistical significance (Fig. 7A). As expected, there was no significant induction of IL-17A-secreting CD4^+^ T cells when heat-killed *S. xylosus* was applied topically onto *Nfkbiz* ^+/−^ and *Nfkbiz* ^−/−^ mice (Figs 7A and S5A). Furthermore, we observed significant increases of pro-inflammatory cytokines (i.e., IL-1β, IFN-γ, TNF-α, and IL-17A) and chemokines (i.e., MCP-1, MIP-1α, RANTES, and KC) in *Nfkbiz* ^−/−^ skin after inoculation of *S. xylosus* (Fig. 7B), which also elevated the levels of IL-1β, TNF-α, MCP-1, MIP-1α, and RANTES in the skin of *Nfkbiz* ^+/−^ mice (Fig. S5B). When these findings are considered together, a single commensal bacterial species (i.e., *S. xylosus*) in the skin has the potential to lead to the differentiation of IL-17A-secreting CD4^+^ T cells and to elicit various pro-inflammatory cytokines and chemokines, which may be involved in the onset of skin inflammation.

## Discussion

In this study, we showed that spontaneous dermatitis developed in *Nfkbiz* ^−/−^ mice along with expansion of IL-17A- and IL-22-secreting CD4^+^ T cells. Pyrosequencing analysis indicated significantly decreased bacterial diversity and drastically expanded pathobiont *S. xylosus* in the skin of *Nfkbiz* ^−/−^ mice. Antibiotic treatment relieved the dermatitis in *Nfkbiz* ^−/−^ mice, accompanied by decreased numbers of IL-17A- and IL-22-secreting CD4^+^ T cells. The introduction of *S. xylosus* to the skin of *Nfkbiz* ^−/−^ mice resulted in the predominant expansion of IL-17A-secreting CD4^+^ T cells. These results demonstrate that the expansion of pathobiont *S. xylosus* under *Nfkbiz*-deficient conditions may be a cause of skin dysbiosis and may be mediated by pathogenic IL-17A-secreting CD4^+^ T cells.

Previous studies described humans with AD and psoriasis that exhibited general pattern dysbiosis in the skin. These subjects had decreased bacterial diversity and altered predominant bacterial species^2, 32^. In this study, we found a similar dysbiotic patterns in *Nfkbiz* ^−/−^ mice with spontaneous dermatitis. Specifically, *S. xylosus* accounted for more than 80% of the resident bacteria in the skin of *Nfkbiz* ^−/−^ mice. *S. xylosus* is a commensal bacterium that inhabits the skin of mammals and occasionally humans^33^, and its ability to cause opportunistic infections has been reported^10–12^. We found a correlation between the expansion of *S. xylosus* and the severity of inflammation in *Nfkbiz* ^−/−^ mice, a finding that is supported by a recent study describing the dominant expansion of *S. aureus* among patients with more severe AD^34^. Moreover, the fact that antibiotics specific to *S. xylosus* resulted in the complete suppression of dermatitis^35, 36^ supports the notion that the dysbiosis provoked by the predominance of *S. xylosus* is closely related to skin inflammation.

AD has been considered an allergic Th2-mediated disease due to the high levels of IgE Ab^37^; however, others suggest that AD is more heterogeneous, given the contributions of other T cells such as Th17 and Th22^16, 38, 39^. Among several subsets of AD, intrinsic AD and Asian AD patients preferentially exhibit the activation of Th17 and Th22 cytokines^40, 41^. These patients show features typical of persons with psoriasis, such as parakeratosis and epidermal hyperplasia^41^. Although the numbers of Th17 and Th22 cells were found to be increased systemically and in the skin of AD patients, the roles of IL-17A and IL-22 in the pathogenesis of this disease remain unclear. In this context, another recent study addressed the possibility that IL-17A acts as an inducer for Th2-type immune responses in murine AD models^42^. On the other hand, IL-22 is known to promote keratinocyte proliferation. IL-23 produced by keratinocytes in response to endogenous TLR4 ligands polarized skin DCs to stimulate the IL-22 response^43^. In our study, we found that IL-17A and IL-22, more than any other cytokines, were significantly reduced in response to the administration of antibiotics (Fig. 5C) and that *S. xylosus* inoculation onto *Nfkbiz* ^−/−^ mice led to elevated IL-17A and IL-22 cytokines in skin homogenates (Fig. 7A). In this context, others demonstrated that the onset of skin inflammation in *Nfkbiz* ^−/−^ is independent of the Th2 response by showing that dermatitis still developed in *Nfkbiz* ^−/−^Stat6^−/−^ mice^26^. We further observed that IFN-γ remained elevated even after antibiotic treatment and improvement of skin inflammation (Figs 4C and S2). These lines of evidence suggest that Th17 and Th22 cells rather than Th1 cells may be directly involved in the development of skin inflammation in *Nfkbiz* ^−/−^ mice.

Our results indicate that the topical association of *S. xylosus* elevates chemokine secretion in the skin (Fig. 7B). Others showed that MCP-1 and MIP-1α participate in both immediate and delayed skin reactions and trigger the recruitment of local T cells, macrophages, and granulocytes^44^. Transgenic mice overexpressing MCP-1 in the epidermis also exhibit a dramatic increase of MHCII^+^ cells in the dermis and show augmentation of the contact hypersensitivity reaction^45^. We speculate that the expansion of *S. xylosus* first stimulates chemokine secretion by keratinocytes and/or fibroblasts, which may be involved in the recruitment of innate immune cells in the skin that are indispensable in the onset of skin inflammation. In support of this possibility, the inoculation of *S. xylosus* also increased migratory DCs (CD11c^+^ MHCII^hi^) in skin-draining lymph nodes (Fig. S6). Other studies indicate that MCP-1 is also responsible for the recruitment of Th17 cells^46^, while IL-17A stimulates fibroblasts to produce MCP-1 and RANTES^47, 48^. Overall, these results imply that the expansion of a single pathobiont such as *S. xylosus* provokes inflammation in local mucosal tissues through the induction of chemokines and Th17 and Th22 responses.

In *Nfkbiz* ^−/−^ mice with severe dermatitis, we found a marked increase in CD103^+^ dDCs, which are rare in normal skin (Fig. 2A). This subset was significantly reduced after antibiotic treatment (Fig. 5A). However, topical inoculation of *S. xylosus* was unable to drive the recruitment of CD103^+^ dDCs in the skin (data not shown). Others found that CD103^+^ DCs in non-lymphoid tissues are functionally specialized in the cross-presentation of antigens to CD8^+^ cells^49^. CD207^+^ dDCs, often referred to as CD103^+^ DCs in the skin, induce CD8^+^ T-cell and Th1-cell responses^15, 50^. However, there have been no reports of CD103^+^ dDCs in skin of *Nfkbiz* ^−/−^ mice with dermatitis, though its presence in steady-state skin-draining lymph nodes is known^51^. Further studies on CD103^+^ dDC function are needed to assess their role in Th17 differentiation during *S. xylosus* infection.

IκBζ, encoded by the *Nfkbiz* gene, is a transcription factor known to promote IL-17A production. For instance, IκBζ^−/−^ mice show resistance to diseases with enhanced Th17 responses, including experimental autoimmune encephalomyelitis (EAE) and psoriasis^19, 52^. However, in this study, we observed robust Th17 responses as well as expression of IL-17A signature genes (i.e., S100a8, S100a9, CAMP, and LCN2) in the skin of *Nfkbiz* ^−/−^ mice with spontaneous dermatitis (Fig. S3). We assume that, unlike RORγt, IκBζ is not a master regulator for Th17 cells. It could thus function as a Th17 cell-specific transcription factor in cooperation with ROR nuclear factors. The overexpression of ROR nuclear factors drove IL-17A production in *Nfkbiz* ^−/−^ T cells^19^. In our study, we detected the upregulation of other transcription factors that contribute to Th17 differentiation, such as BATF, which remodels chromatin to enable the access of transcription factors to Th17-specific loci^53^; Hif-1α, which can directly increase RORγt expression and drive IL-17A expression with RORγt^54^; and IL-23A, which is essential for the induction of pathogenic Th17 cells^55^ in the skin of *Nfkbiz* ^−/−^ mice with dermatitis (Fig. S3). These results provide support for a molecular mechanism of an enhanced Th17 response in *Nfkbiz* ^−/−^ mice.

In conclusion, our findings demonstrate that *S. xylosus* is an important pathobiont that accelerates dermatitis in *Nfkbiz*-deficient mice. We also found that a single commensal bacterium can modulate innate and acquired immune responses in the skin. Although the commensal microbiota maintains a symbiotic relationship with the host immune system and plays a beneficial role in the host under steady-state conditions, it can provoke and trigger inflammation under *Nfkbiz*-deficient conditions. These results suggest that the *Nfkbiz* gene has a potential regulatory role in the microbiota-skin immunity axis.

## Methods

### Ethics statement

All animal experiments were approved by the Institutional Animal Care and Use Committee of Asan Biomedical Research Center (Approval No. PN 2014-12-063) and the studies were carried out in accordance with the approved guidelines and regulations. All experiments were performed under anesthesia with a mixture of ketamine (100 mg/kg) and xylazine (20 mg/kg), and all efforts were made to minimize suffering.

### Mice

*Nfkbiz* ^−/−^ mice were provided by Prof. Shizuo Akira (IFReC, Osaka University, Japan). All mice were maintained under specific pathogen-free conditions in the animal facility at Asan Biomedical Research Center (Seoul, Korea), where they received sterilized food and water *ad libitum*.

### Clinical score

Pathological phenotypes of mice were scored according to the criteria as described previously^24^, with slight modification: (1) swelling of the eyelid; (2) periocular skin erosion and eyes with discharge that were difficult to open; (3) inflammation reaching the skin of the ear and part of the skin on the body; (4) erosion and loss of hair in the whole body; and (5) severe erosion and loss of hair in the whole face and body.

### Preparation of mononuclear cells from skin

Mouse trunk skin was shaved, peeled off, and placed epidermal side down in a Petri dish. Subcutaneous fat tissue and blood vessels were scrapped off with a Scalpel (Feather, Osaka, Japan). Skin tissue was then incubated with 0.25% trypsin (Gibco, Waltham, MA) at 37 °C for 2 h. Epidermal cells were separated from the dermis by mechanical separation and vigorously agitated in RPMI 1640 (Gibco) containing 10% FBS (Gibco) at 37 °C for 10 min. The dermis was then minced with scissors and further treated with 2.5 mg/ml collagenase IV (Sigma Aldrich, St. Louis, MO) and 0.2 mg/ml DNase (Roche) at 37 °C with stirring. After 1 h, digestion was stopped by the addition of 10 mM EDTA. Epidermal and dermal cell suspensions were filtered and enriched for immune cells using a 40–75% Percoll density gradient (GE Healthcare, Buckinghamshire, UK).

### Flow cytometry analysis and antibodies

For cytokine analysis, cell suspensions were cultured in RPMI 1640 medium supplemented with 10% FBS and 1% penicillin and streptomycin (Gibco) with 50 ng/ml PMA (Sigma Aldrich) and 1 μg/ml ionomycin (Sigma Aldrich) in the presence of Brefeldin A (BD Biosciences, Franklin Lakes, NJ) overnight at 37 °C. Cells were stained with Live/Dead Cell Stain kit (Invitrogen, Carlsbad, CA) and surface markers after treatment with purified anti-mouse CD16/32 Ab (BD Biosciences). Fixation and permeabilization were performed with BD Cytofix/Cytoperm (BD Biosciences), followed by intracellular cytokine staining. The following Abs from BD Biosciences, eBioscience (Santa Clara, CA), or BioLegend (San Diego, CA) were used for analysis: anti-CD45 (30-F11), anti-CD4 (RM4–5), anti-CD8α (53–6.7), anti-TCRγδ (GL3), anti-IFN-γ (XMG1.2), anti-IL-4 (11B11), anti-IL-17A (TC11-18H10), anti-IL-22 (IL-22JOP), anti-MHC II (M5/114.15.2), anti-CD207 (4C7), anti-CD11b (M1/70), anti-CD11c (HL3), and anti-CD103 (2E7). Cell acquisition was performed on a BD Biosciences FACSCanto II flow cytometer and data were analyzed using FlowJo software (Tree Star, Ashland, OR).

### 16s rRNA gene pyrosequencing analysis

Genomic DNA was extracted from skin tissue using the NucleoSpin Soil kit (Macherey-Nagel, Düren, Germany). PCR amplification was performed using primers targeting the segment from the V1 to V3 regions of the 16S rRNA gene with extracted gDNA. For bacterial amplifications, we used barcoded primers of 9 F (5′-CCTATCCCCTGTGTGCCTTGGCAGTC-TCAG-AC-AGAGTTTGATCMTGGCTCAG-3′; the underlined sequence indicates the target region primer) and 541 R (5′-CCATCTCATCCCTGCGTGTCTCCGAC-TCAG-X-AC-ATTACCGCGGCTGCTGG-3′; X indicates the unique barcode for each subject). The amplifications were performed under the following conditions: initial denaturation at 95 °C for 5 min, followed by 30 cycles of denaturation at 95 °C for 30 sec, primer annealing at 55 °C for 30 sec, and extension at 72 °C for 30 sec, with final elongation at 72 °C for 5 min. The amplified products were purified with the QIAquick PCR purification kit (Qiagen, Valencia, CA). Equal concentrations of purified products were pooled together and short fragments (non-target products) were removed with an AMPure bead kit (Agencourt Bioscience, Beverly, MA). The quality and product size were assessed on a Bioanalyzer 2100 (Agilent, Palo Alto, CA) using a DNA 7500 chip. Mixed amplicon sequencing was performed by emulsion PCR and then deposited on picotiter plates. The sequencing was carried out at Chunlab (Seoul, Republic of Korea) on a GS Junior Sequencing System (Roche, Branford, CT), in accordance with the manufacturer’s instructions.

### Microarray analysis

RNA from skin tissues was isolated with TRIzol (Invitrogen) reagent. RNA labeling and hybridization were performed by using the Agilent one-color microarray-based gene expression analysis kit (Agilent Technology, Santa Clara, CA). In brief, total RNA (200 ng) from each sample was linearly amplified and labeled with Cy3-dCTP. The labeled cRNAs were purified by RNAeasy mini kit (Qiagen). Each labeled cRNA (600 ng) was fragmented by adding blocking agent and fragmentation buffer, and then heated at 60 °C for 30 min. Finally GE hybridization buffer was added to dilute the labeled cRNA. Hybridization solution was dispensed into the gasket slide and assembled with the Agilent SurePrint G3 Mouse GE 8X60 K kit. The slides were incubated for 17 h at 65 °C in an Agilent hybridization oven and then washed. The hybridized array was immediately scanned with an Agilent Microarray Scanner D.

### IPA (Ingenuity Pathway Analysis)

IPA (Ingenuity Systems, http://www.ingenuity.com) was used for gene network analysis, which was carried out to identify the most significant gene sets associated with disease process and molecular and cellular functions in significantly altered genes (described in the Figs 6 and S3). The significance of over-represented gene sets was estimated by the right-tailed Fisher’s exact test. Gene network analysis was carried out by using a global molecular network developed from information contained in the Ingenuity Knowledge Base. Identified gene networks were ranked according to scores provided by IPA.

### Antibiotic treatment

Mice were treated with 0.5 mg/ml cephalexin and enrofloxacin (Sigma Aldrich) in drinking water.

### Bacterial isolation and inoculation onto mice

To isolate *S. xylosus* from skin, swabs from the shaved back skin of mice (1 cm^2^ or the infection area) were placed in PBS and cultured on MSA (BD Biosciences). The colonies from the plates were sequenced by Macrogen (Seoul, Republic of Korea). *S. xylosus* was cultured in mannitol salt broth (BD Biosciences) at 37 °C overnight in a shaking incubator. Bacteria were enumerated by assessing the colony-forming units (CFUs) using a standard plate count method and by measuring optical density at 600 nm. For the topical inoculation of bacteria, a bacterial suspension (10^9^ CFUs) in PBS was topically applied to the shaved back skin of mice using a sterile cotton swab. Bacteria were applied every other day.

### Histological analysis and immunofluorescence microscopy

After sacrifice, mouse back skin was fixed in 4% paraformaldehyde, embedded in paraffin, and stained with hematoxylin-eosin. Microscopic images were obtained (BX53; Olympus Optical, Tokyo, Japan). For immunofluorescence microscopy, skin tissues were frozen, embedded in OCT compound, sectioned, and fixed with −20 °C acetone for 5 min. Slides were blocked with PBS containing 1% BSA for 1 h at room temperature and stained with anti-CD8α (53–6.7), anti-CD4 (RM4-5), anti-CD11b (M1/70), anti-CD207 (4C7), and anti-CD11c (HL3) Abs (BD Biosciences) overnight at 4 °C. Tissues were washed and stained with 4′,6-diamidino-2-phenylindole (DAPI; BD Pharmingen) for 2 min at room temperature, followed by mounting with PermaFluor mountant (Thermo, Waltham, MA). Images were captured on an LSM 710 confocal microscope (Carl Zeiss, Oberkochen, Germany).

### IgE and cytokine analysis

Serum total IgE Ab level was determined using mouse IgE ELISA kit (BD Biosciences). Supernatant was collected after skin tissues were weighed and homogenized with Tris-EDTA buffer (10 mM Tris-HCl and 1 mM EDTA, pH 7.4, 0.05% sodium azide, 1% Tween-80, protease inhibitor cocktail) and centrifuged at 11,000 × g for 10 min at 4 °C. Cytokines and chemokines from tissue homogenates were measured by a ProcartaPlex Multiplex immunoassay kit (eBioscience), in accordance with the manufacturer’s instructions.

### Trans-epidermal water loss

TEWL was measured by VapoMeter SWL4001JT (Delfin, Kuopio, Finland).

### Statistics

GraphPad Prism software (GraphPad, La Jolla, CA) was used for statistical analysis. Significant differences between two groups were analyzed with two-tailed unpaired *t*-test. Multiple groups were analyzed by two-way ANOVA followed by Bonferroni’s *post hoc* test (*p < 0.05; **p < 0.01).

## Electronic supplementary material

Supplemental Information 
