# Supplementary material for: The resident pathobiont Staphylococcus xylosus in Nfkbiz-deficient skin accelerates spontaneous skin inflammation
Source: Sci Rep. 2017 Jul 24;7:6348. doi: 10.1038/s41598-017-05740-z (PMC5524713; doi:10.1038/s41598-017-05740-z)
Supplement: Supplementary file 1 — Supplemental Information [file 41598_2017_5740_MOESM1_ESM.pdf]

The resident pathobiont *Staphylococcus xylosus* in *Nfkbiz*-deficient skin accelerates spontaneous skin inflammation

Yeji Kim[1], Yong-Soo Lee[1], Jin-Young Yang[1], Su-Hyun Lee[1], Yun-Yong Park[2], and Mi-Na Kweon[1*]

[1]*Mucosal Immunology Laboratory, Department of Convergence Medicine, University of Ulsan College of Medicine/Asan Medical Center, Seoul, Republic of Korea*

[2]*ASAN Institute for Life Science, Asan Medical Center, Seoul, Republic of Korea*

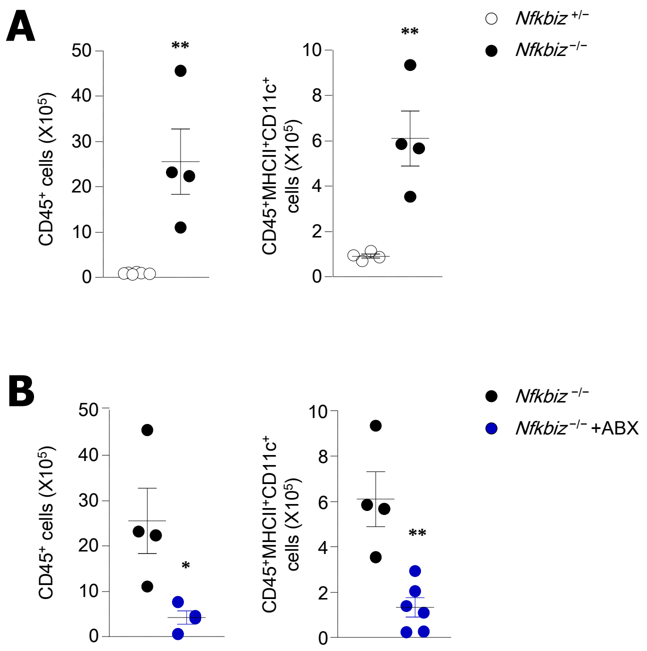

Fig E1. **Accumulation of immune cells in skin of *Nfkbiz*$^{-/-}$ mice with dermatitis is reduced by ABX treatment**. (**A**) FACS analysis of $CD45^+$ cells and $CD45^+MHCII^+CD11c^+$ cells in the skin of *Nfkbiz*$^{+/-}$ and *Nfkbiz*$^{-/-}$ mice. (**B**) FACS analysis of $CD45^+$ cells and $CD45^+MHCII^+CD11c^+$ cells in the skin of untreated and ABX-treated *Nfkbiz*$^{-/-}$ mice. Data are representative of three independent experiments. $*p < 0.05$, $**p < 0.01$.

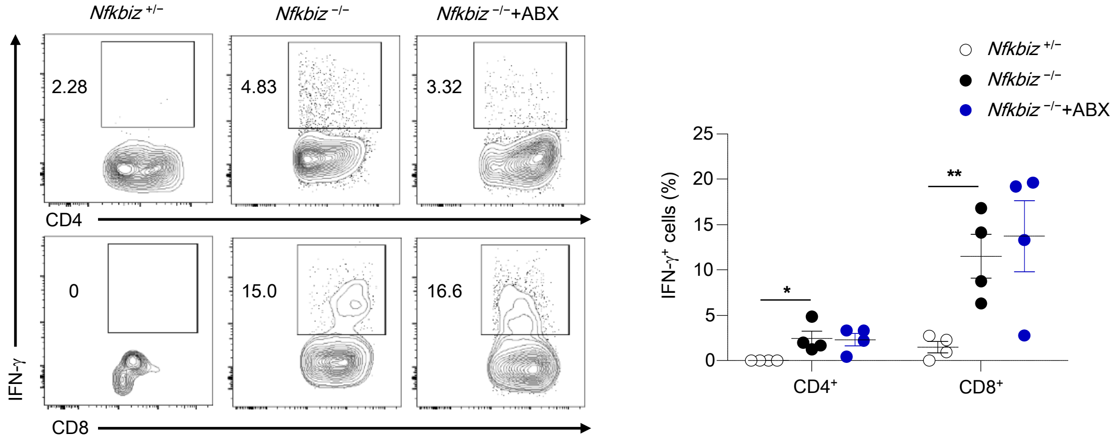

Fig E2. **The level of IFN-γ in the skin of *Nfkbiz*$^{-/-}$ mice remains unchanged after ABX treatments.** FACS analysis of IFN-γ production by CD4^{+} and CD8^{+} cells in the skin of *Nfkbiz*$^{+/-}$, untreated, and ABX-treated *Nfkbiz*$^{-/-}$ mice. Data are representative of three independent experiments. *$p < 0.05$, **$p < 0.01$.

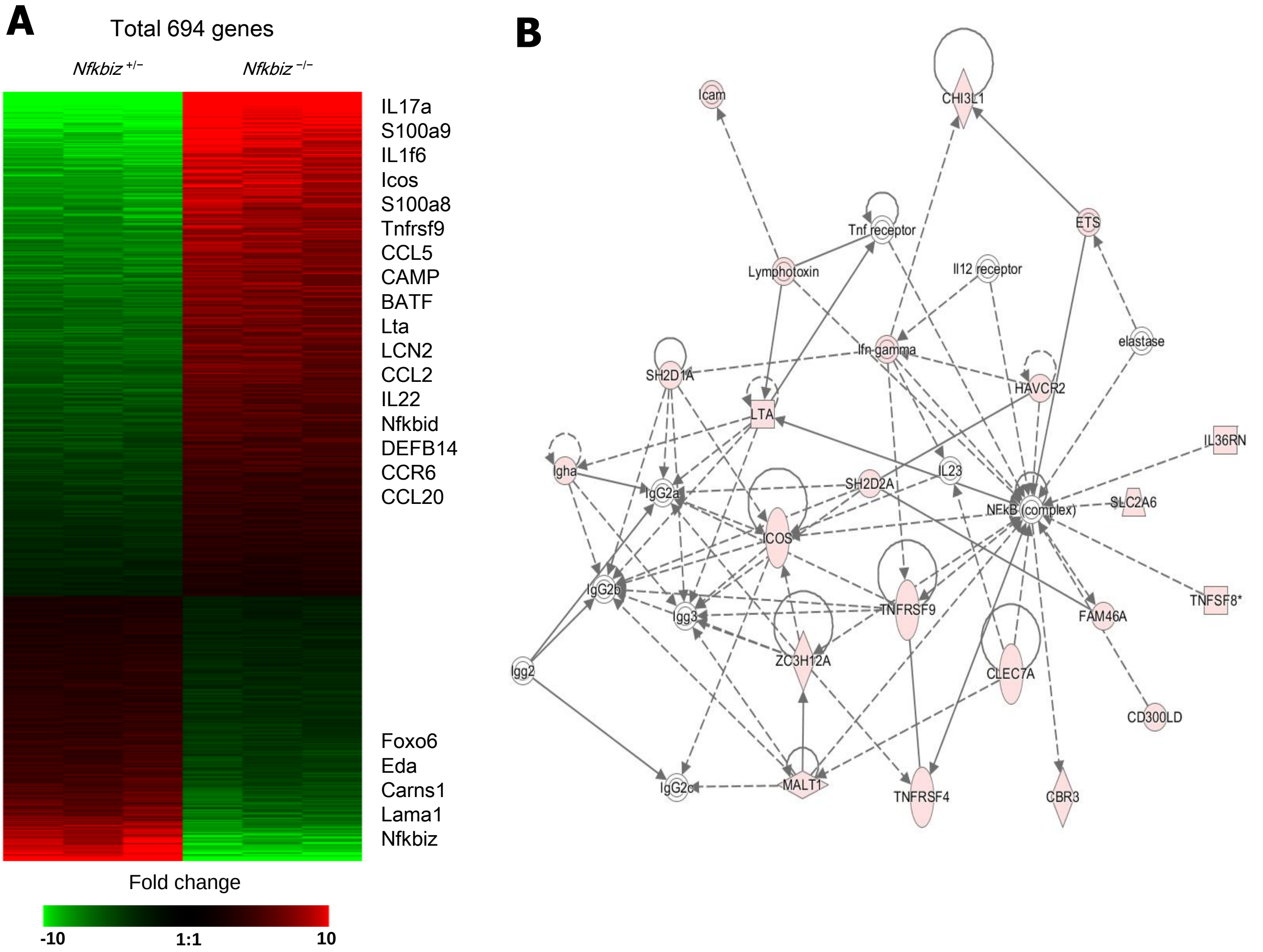

Fig E3. **Gene expression related to inflammation is upregulated in the skin of *Nfkbiz*$^{-/-}$ mice.** (**A**) Microarray analysis of the skin from *Nfkbiz*$^{+/-}$ and *Nfkbiz*$^{-/-}$ mice (n = 3 per group). Genes exhibiting significant change in expression (fold change > 1.0 and $p < 0.05$) between *Nfkbiz*$^{+/-}$ and *Nfkbiz*$^{-/-}$ mice were clustered in a heatmap. (**B**) A gene interaction network, associated with spontaneous dermatitis in *Nfkbiz*$^{-/-}$ mice, was established using Ingenuity Pathway Analysis (IPA) software. Solid lines indicate direct interaction, while dashed lines indicate indirect interaction.

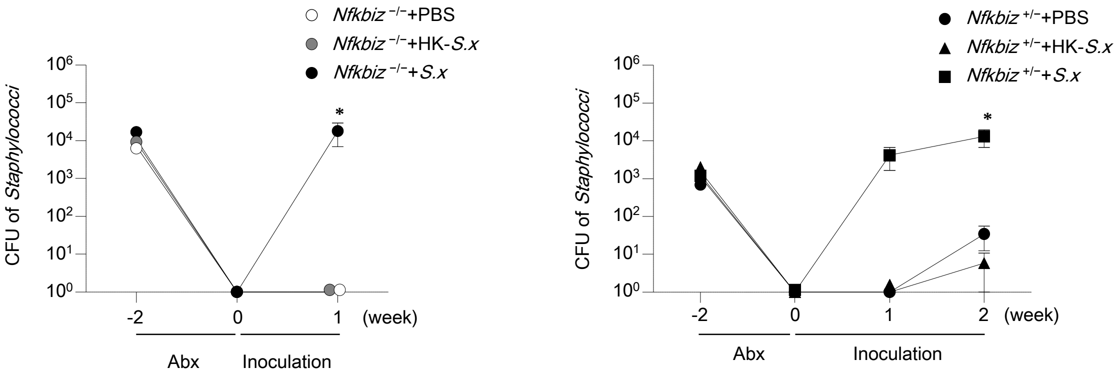

Fig E4. **Colonization pattern of *S. xylosus* after its topical application is shown.** CFUs of *Staphylococcus* species of skin swabs from *Nfkbiz*$^{-/-}$ and *Nfkbiz*$^{+/-}$ mice upon the inoculation of PBS, heat-killed *S. xylosus* (HK-*S.x*), and *S. xylosus* (*S.x*). Data are mean ± s.e.m. Data are representative of two independent experiments. *$p < 0.05$, **$p < 0.01$.

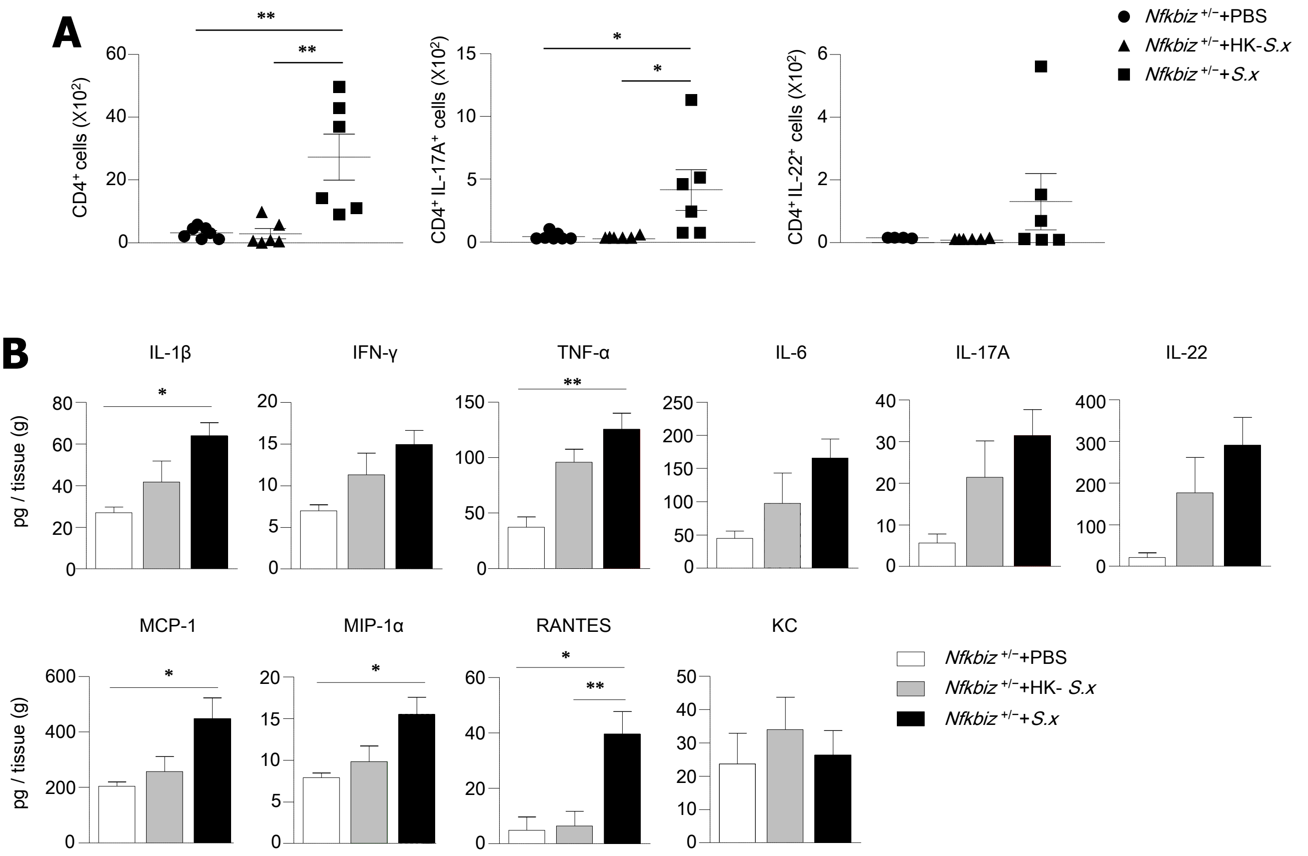

Fig E5. **Inoculation of *S. xylosus* triggered the differentiation of Th17 cells and the production of cytokines and chemokines in *Nfkbiz*$^{+/-}$ mice.** (**A**) FACS analysis of the IL-17A production by CD4^{+} cells in the skin of *Nfkbz*$^{+/-}$ mice with the inoculation of PBS, heat-killed *S. xylosus* (HK-*S.x*), and *S. xylosus* (*S.x*). (**B**) Cytokine and chemokine levels in skin homogenate from *Nfkbiz*$^{+/-}$ mice with the inoculation of PBS, HK-*S.x*, and *S.x*. Data are mean ± s.e.m. Data are representative of three independent experiments. *$p < 0.05$, **$p < 0.01$.

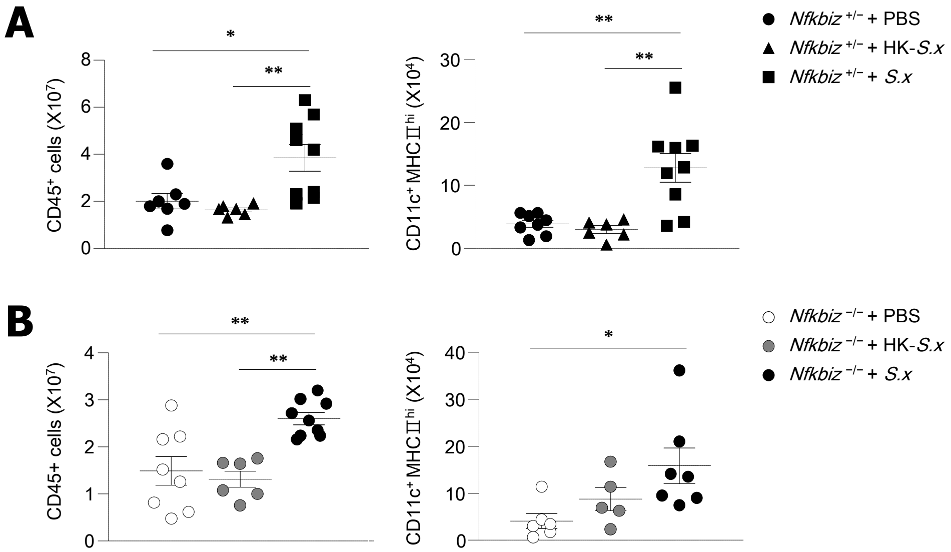

Fig E6. **Cells in skin draining lymph nodes (sdLNs) from *Nfkbiz*$^{-/-}$ and *Nfkbiz*$^{+/-}$ mice were increased after the inoculation of *S. xylosus*.** (**A**) FACS analysis of CD45$^+$ cells and CD45$^+$MHCII$^+$CD11c$^+$ cells in sdLNs from *Nfkbiz*$^{+/-}$ mice with the inoculation of PBS, HK-*S.x*, and *S.x*. (**B**) CD45$^+$ cells and CD45$^+$MHCII$^+$CD11c$^+$ cells in sdLNs from *Nfkbiz*$^{-/-}$ mice with the inoculation of PBS, HK-*S.x*, and *S.x*. Data are representative of three independent experiments. $*p < 0.05$, $**p < 0.01$.
